# Comparative Diagnostic Efficacy of HeartLogic and TriageHF Algorithms in Remote Monitoring of Heart Failure: A Cohort Study

**DOI:** 10.3390/jcdd12060209

**Published:** 2025-05-31

**Authors:** David Ledesma Oloriz, Daniel García Iglesias, Rodrigo Ariel di Massa Pezzutti, Fernando López Iglesias, José Manuel Rubín López

**Affiliations:** 1 Arrhythmia Unit, Cardiology Department, Hospital Universitario Central de Asturias, 33011 Oviedo, Spain; 2 Instituto de Investigación Sanitaria del Principado de Asturias (ISPA), 33011 Oviedo, Spain

**Keywords:** heart failure, implantable cardiac defibrillator, TriageHF, Heart Logic

## Abstract

Introduction: Implantable defibrillator devices (ICDs) can be used for remote monitoring of different variables, including some related to Heart Failure (HF). Two different algorithms (TriageHF and HeartLogic) arise by combining some of these variables to generate an estimation of HF decompensation risk in the following days. Until now, no other trial has evaluated both algorithms in a head-to-head comparison. The primary objective is to compare diagnostic accuracy of both algorithms in a similar cohort of patients. Material and Methods: Descriptive monocentric cohort study of a series of 64 patients who have been implanted with a Medtronic or Boston Scientific ICD with the TriageHF or Heart Logic algorithm available during the period between January 2020 and June 2022, with a total of 27 patients in the HeartLogic group and 37 patients in the TriageHF group. Results: During the period of the study there were a total of 1142 alarms analyzed. There were no differences in the basal characteristics of both groups. We reported a risk alarm–patient ratio of 1.31 ± 1.89 in the HeartLogic group and of 3.32 ± 3.08 in the TriageHF group (*p* < 0.01). In the TriageHF group, we reported a lower specificity with (0.76), with higher sensitivity (0.97) and PPV (0.18), and similar NPV (1). Survival analysis shows no statistical differences between both algorithms in the 30 days following the alert. Conclusions: TriageHF algorithm had higher sensibility and PPV, leading to a higher number of alerts/patients, while HeartLogic algorithm had a better specificity. These differences should be considered to optimize patient follow-ups in home monitoring.

## 1. Introduction

Despite the development of new therapeutic approaches in the treatment of patients with heart failure (HF), morbidity and mortality rates can still be improved [1]. The course of this syndrome intercalates periods of clinical stability with decompensation. These decompensations correlate well with morbidity and mortality, quite often requiring hospital admission for intensive treatment [2]. Traditionally, remote management strategies for patients with heart failure have included weight monitoring and fluid intake control. With the integration of telecommunication technologies into routine clinical practice, more sophisticated methods have emerged to support the remote monitoring of patients with heart failure. Although these new technologies have demonstrated diagnostic capability, the cost-effectiveness of their implementation within healthcare systems remains to be established [3].

According to the most recent clinical guidelines, implantable defibrillator devices (ICDs) are indicated in the treatment of patients with HF and left ventricular dysfunction to reduce their mortality [4]. Besides the reduction in mortality, these devices can be used for remote monitoring of different variables, including some related to HF. Two different algorithms (TriageHF and HeartLogic) arise by combining some of these variables to generate an estimation of HF decompensation risk in the following days [5,6]. HeartLogic uses a quantitative approach, integrating variables like intrathoracic impedance and activity level to predict heart failure risk, with a value of 16 or higher indicating increased risk of heart failure. TriageHF, on the other hand, follows a qualitative approach, integrating variables such as the OptiVOL fluid index and heart rate variability. It estimates heart failure risk as low, moderate, or high within the next 30 days. The main difference between the two algorithms lies in the way they estimate the risk of decompensation. The TriageHF algorithm provides different risk levels, while HeartLogic assigns a threshold value above which the risk of heart failure increases [7,8,9,10].

Different studies have been carried out to prove the utility of both algorithms for remote monitoring of patients with HF. In general, they have proven to have high negative predictive values with variable specificity and sensibility [7,8,10,11,12]. Until now, no other trial has evaluated both algorithms in a head-to-head comparison. There are other algorithms based on a combination of variables, such as HeartInsight and CorVue, although they are supported by fewer studies [13].

### Objective

Primary Objective: The primary objective was to describe the performance of both algorithms in a cohort of patients with heart failure.

Secondary Objective: The secondary objective was to compare both algorithms by identifying potential differences in their diagnostic capabilities, with particular attention to the number of false positive alarms.

## 2. Methods

### 2.1. Study Population

This study included all patients implanted with a Medtronic (Minneapolis, MN, USA) Amplia ICD-TRC or Visia ICD (with the TriageHF algorithm activated) or a Boston Scientific (Marlborough, MA, USA) Resonate ICD-TRC or Resonate ICD (with the HeartLogic algorithm activated) between January 2020 and June 2022. All patients had a prior history of symptomatic heart failure (NYHA class II–IV) and met the clinical indications for ICD implantation (with or without CRT) based on current clinical recommendations [3]. Eligible patients were required to be older than 18 years and enrolled in a home monitoring program (Medtronic Carelink or Boston Scientific Latitude) to facilitate HF algorithm implementation.

### 2.2. Data Adquisition and Follow up

Clinical characteristics were recorded at the time of device implantation. Follow-up visits were conducted in person at 1 month post-implantation and subsequently every 12 months. At the conclusion of the study, a final telephone follow-up was conducted, and medical records were comprehensively reviewed. Prospective follow-up ended in January 2023. A heart failure (HF) event was defined as a clinical episode of congestion requiring intravenous loop diuretic therapy in the emergency department or hospitalization. A risk alert was defined as an algorithm-generated alert persisting for more than 7 consecutive days. A positive alert was considered concordant with a heart failure event when the event occurred within 30 days following a heart failure risk alert. We considered that as a true positive alarm. If no heart failure occurred within the subsequent 30 days after the alert, the alert was classified as a false positive.

### 2.3. Ethical Considerations

The study was conducted in accordance with the ethical principles outlined in the Declaration of Helsinki. The research protocol received approval from the institution’s ethics committee, and all relevant ethical guidelines and regulations were followed throughout the study.

### 2.4. Statistical Analysis

Continuous variables are expressed as mean ± standard deviation and were compared using Student’s *t*-test (following an assessment of normality). Categorical variables are presented as percentages and compared using the Chi-square test (with Yates correction applied where necessary).

Kaplan–Meier survival analysis was performed to evaluate the incidence of heart failure within 30 days following a risk alert. Comparisons of survival free of HF events after an alert were conducted using the Log-Rank test.

Statistical analyses were performed using R software with 4.1 version (R Foundation for Statistical Computing, Vienna, Austria). A *p*-value < 0.05 was considered statistically significant throughout the analysis.

## 3. Results

### 3.1. Population Characteristics

The study included 64 patients in total, with 27 in the HeartLogic group and 37 in the TriageHF group. The baseline characteristics of the study population were similar across both groups, with no significant differences in terms of age, gender, comorbidities, or previous treatment. Key clinical characteristics included an average age of 65 years, with 79% of patients being male, and similar rates of comorbidities such as ischemic cardiomyopathy and chronic kidney disease (see Table 1 for detailed clinical data).

### 3.2. Algorhythm Alert Summary

A total of 1142 alarms were analyzed during the study period, of which 533 were generated by HeartLogic and 609 by TriageHF. Of these 1142 alarms, 168 were heart failure risk alarms. We reported a risk alarm–patient ratio of 1.31 ± 1.89 in the HeartLogic group and of 3.32 ± 3.08 in the TriageHF group (*p* < 0.01).

HeartLogic arm patients were less time at risk of HF than the patients of the TriageHF arm (5.65% VS. 19.55%; *p* < 0.01), showing less clinical HF episodes too, with a HF episode-patient rate of 0.115 ± 0.325 in the HeartLogic group vs. 0.837 ± 1.726 in the TriageHF group (*p* = 0.017) with a total number of 34 heart failure episodes.

### 3.3. Algorithm Performance

In the HeartLogic group, we observed a sensitivity of 0.67, a specificity of 0.94, a positive predictive value (PPV) of 0.06 and a negative predictive value (NPV) of 1. In the TriageHF, we reported a lower specificity (0.76), with higher sensitivity (0.97) and PPV (0.18), and similar NPV [1]. We also observed a false positive-patient rate of 1.27 (±1.77) in the Heart Logic group; meanwhile, we found a rate of 3.56 (±3.42) in the TriageHF group (*p* < 0.001). A more detailed algorithm performance can be found in Table 2.

**Figure 1:**
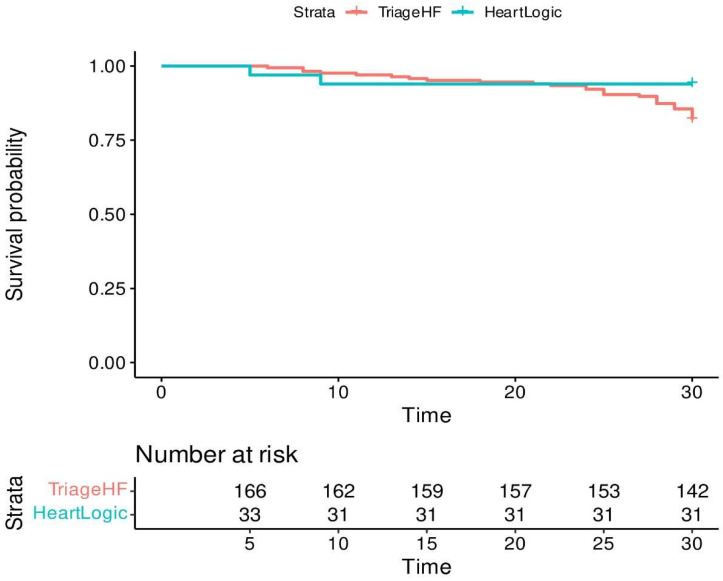
Survival analysis for the 30 days after each alert.

Figure 1: Survival analysis comparing both algorithms.

Survival analysis (Figure 1) shows no statistical differences between both algorithms in the 30 days following the alert; nevertheless, there is a tendency in HeartLogic alarms to result in an HF episode within fewer days from the alarm onset.

## 4. Discussion

This study is the first designed for a comprehensive analysis comparing the most widely used remote monitoring algorithms for HF on ICDs (HeartLogic and TriageHF). Both algorithms predict HF decompensation events, potentially improving patient management and reducing hospital admissions. Previous studies based on both algorithms have tested a wide range of strategies, varying in event definition criteria, patient selection, and intervention protocols. As a result, highly variable outcomes have been reported in terms of sensitivity, specificity, and predictive values. However, a common finding across studies is that both algorithms exhibit high false-positive rates [6,7,10,11,14,15,16,17,18,19].

Our results reveal significant differences in performance between both algorithms. The HeartLogic group had a significantly lower number of heart failure episodes, with no differences found in the baseline characteristics between the groups. A potential hypothesis for this finding could be differences in the initiation of SGLT2 inhibitors or in the loop diuretic prescription. These differences should be considered in daily clinical practice, as an algorithm with high sensitivity may lead to a greater number of alerts but a lower risk of inadequately diagnosed episodes of HF. On the other hand, a more specific algorithm may reduce the number of alerts, but this could entail a relative risk of not diagnosing certain episodes of HF. Moreover, it must be considered that these algorithms are used in a home monitoring context, and an excessive number of false positive alerts can lead to an excessive workload from a resource management perspective.

In our work, TriageHF algorithm had higher sensitivity and PPV compared with HeartLogic, which had a higher specificity and fewer false positive alarms. Moreover, TriageHF algorithm had a higher number of alerts/patients compared to Hart Logic algorithm.

The findings of this study may be limited by the sample size as well as by the imbalance in the number of heart failure episodes between groups, which could partially influence the results. However, despite the small sample size, we consider that the number of low-risk alerts recorded in the study is sufficient to strongly support the observed differences in false positive rates between the two algorithms.

## 5. Conclusions

TriageHF and HeartLogic have distinct diagnostic profiles that can influence patient management in the context of remote HF monitoring. TriageHF has higher sensitivity and PPV, resulting in a greater number of alarms, whereas HeartLogic has higher specificity, resulting in fewer false-positive alerts. These findings suggest that the choice of algorithm should be tailored to individual patient needs, balancing the benefits of early detection against the risk of false positives. Further studies are needed to validate these findings in larger, multicentric cohorts and to explore the impact of these algorithms on clinical outcomes such as hospitalization and mortality.

## Figures and Tables

**Table 1:** Comparison of basal characteristics and algorithm features between both groups.

Variable	Total (n = 64)	Boston (n = 27)	Medtronic (n = 37)	*p*-Value
Male sex	51 (79%)	21 (77%)	30 (81%)	0.746
Mean age at implantation	65.22 (±11.35)	67.07 (±11.2)	63.86 (±11.407)	0.267
CRT-ICD	57 (89%)	26 (96%)	31 (83%)	0.113
Ischemic cardiomyopathy	27 (42%)	11 (41%)	16 (43%)	0.618
Primary prevention implantation	51 (79%)	22(81%)	29 (78%)	0.761
Arterial hypertension	42 (65%)	18 (66%)	24 (65%)	0.881
Diabetes mellitus	20 (31.2%)	9 (33%)	12 (32.4%)	0.494
Chronic kidney disease	17 (26.5%)	9 (33%)	7 (19%)	0.295
Dyslipidemia	34 (53.1%)	14 (51.8%)	19 (51.3%)	0.688
Active tabaquism	8 (12.5%)	4 (14.8%)	4 (10.81%)	0.452
Previous smoking	39 (61%)	18 (66%)	21 (58%)	0.280
Previous cpod	9 (14%)	4 (14.8%)	5 (13.5%)	0.882
Atrial fibrillation	20 (31.25%)	10 (37%)	10 (27%)	0.695
Permanent atrial fibrillation	16 (25%)	8 (29.6%)	8 (21.6%)	0.7
Previous Left Ventricle Ejection Fraction				
• >53%	4 (6.25%)	0 (0%)	4 (10.8%)	0.175
• 40–50%	2 (3.12%)	1 (3.7%)	1 (2.7%)	
• 30–40%	13 (20.31%)	8 (29.6%)	5 (13.5%)	
• <30%	44 (68.75%)	18 (66%)	26 (70.27%)	
Previous Treatment				
• BETABLOCKERS	52 (81%)	22(81.4%)	30 (81%)	0.84
• ACE-I/ARA-II	29 (45%)	13 (48%)	16 (43%)	0.9
• MRA	29 (45%)	11 (40.7%)	18 (49%)	0.291
• ARNI	22 (34%)	8 (29.6%)	14 (37.8%)	0.358
• SGLT1-I	14 (21.8%)	3 (11.1%)	11 (28%)	0.122
Previous NYHA Functional Class				
• I	2 (3.12%)	0 (0%)	2 (5.4%)	0.292
• II	44 (68.75%)	17 (63%)	27 (72.9%)	
• III	15 (23.4%)	9 (33%)	6 (16.2%)	
• IV	3 (4.6%)	1 (4%)	2 (5.4%)	
Clinical Follow-Up				
MEAN FOLLOW-UP (DAYS ± SD)	571.1 (±232.56)	679.65 (±55.4)	494.8 (±276.6)	0.00031
CLINICAL HF EPISODES/PATIENT (MEAN ± SD)	0.539 (±1.378)	0.115 (±0.325)	0.837 (±1.726)	0.017
TIME IN RISK (DAYS ± SD)	82.85 (±132.44)	40.34 (±64.80)	112.72 (±158.33)	0.0157
TIME IN RISK (% OF FOLLOW UP)	13.81 (±17.9)	5.65 (±8.49)	19.55 (±20.36)	<0.001

ACE-I: Angiotensin-Converting Enzyme Inhibitor; ARNI: Angiotensin Receptor–Neprilysin Inhibitor; CRT-ICD: Cardiac Resynchronization Therapy with Implantable Cardioverter-Defibrillator; HF: Hear Failure. MRA: Mineralocorticoid Receptor Antagonist; NYHA: New York Heart Association; SGLT1-I: Sodium-Glucose Cotransporter 1 Inhibitor.

**Table 2:** Comparison of Alarm Frequency and false positive rates Between HeartLogic and TriageHF Algorithms.

	Total	Heart Logic	Triage HF	*p*
Total number of alarms	1142	533	609	
Number of positive alarms	168	33	135	
Number of risk alerts/patient	2.49 (±2.82)	1.31(±1.89)	3.32 (±3.08)	<0.001
False positive-patient rate (mean ± sd)	2.61 (±30.6)	1.27(±1.77)	3.56 (±3.42)	<0.001

## Data Availability

The original contributions presented in this study are included in the article. Further inquiries can be directed to the corresponding author(s).
